# Awareness of cytomegalovirus and risk factors for susceptibility among pregnant women, in Montreal, Canada

**DOI:** 10.1186/s12884-016-0844-9

**Published:** 2016-03-15

**Authors:** Sarah Wizman, Valérie Lamarre, Lena Coic, Fatima Kakkar, Jean-Baptiste Le Meur, Céline Rousseau, Marc Boucher, Bruce Tapiero

**Affiliations:** Infectious Disease Division, Department of Paediatrics, CHU Sainte Justine, Université de Montréal, 3175 Ch. de la Cote-Sainte-Catherine (bureau 7412), Montréal, H3T1C5, QC Canada; Department of Social and Preventive Medicine, Faculty of Medicine, Laval University, Pavillon Ferdinand-Vandry (bureau 4633), 1050 Av de la Medicine, Québec, G1V0A6 QC Canada; Department of Microbiology, CHU Sainte-Justine, Université de Montréal, 3175 Ch. de la Cote-Sainte-Catherine (bureau 2901), Montréal, H3T1C5, QC Canada; Obstetric and Gynecology Department, CHU Sainte Justine, Université de Montréal, 3175 Ch. de la Cote-Sainte-Catherine (bureau 4822), Montréal, H3T1C5, QC Canada; 5632 Irving Layton, Montréal, H4W0A2, QC Canada

**Keywords:** Cytomegalovirus, Serostatus, Awareness, Socio-demographic risk factors, Pregnant women

## Abstract

**Background:**

Advances in diagnostic and therapeutic modalities for congenital cytomegalovirus (CMV) infection have generated a mounting interest in identifying mothers susceptible to CMV. The objectives of this study were to evaluate the prevalence and socio-demographic determinants of CMV susceptibility and CMV awareness, among pregnant women, in Montreal, Quebec.

**Methods:**

Between April and December 2012, women delivering at Centre Hospitalier Universitaire Sainte Justine were recruited for the study. Stored serum from the first trimester of pregnancy was tested for CMV IgG. Knowledge about CMV and socio-demographic characteristics were collected via standardized questionnaire.

**Results:**

Four hundred and ninety one women were enrolled in the study. Overall, 225 mothers (46 %) were seronegative for CMV, and 85 % (*n* = 415) were unaware of CMV or the associated risks in pregnancy. Significant risk factors for CMV seronegative status included Canadian vs. foreign born (aOR 6.88, 95 % CI 4.33–10.94), and high vs. low family income (aOR 4.68, 95 % CI 2.09–10.48). Maternal employment status was the only significant predictor of CMV unawareness, with unemployed mothers at the highest risk (aOR 85.6, 95 % CI 17.3–421.3).

**Conclusions:**

Nearly half of pregnant women studied were at risk of primary infection, and yet, the majority was unaware of potential risks associated with CMV. Canadian born mothers and those with a high socioeconomic status were more likely to be CMV seronegative. Increased education about CMV infection, through public health interventions and obstetrician/pediatric counseling, is needed for all pregnant women.

**Electronic supplementary material:**

The online version of this article (doi:10.1186/s12884-016-0844-9) contains supplementary material, which is available to authorized users.

## Background

Cytomegalovirus (CMV) is the most common cause of congenital infection, with an overall birth prevalence in the developed world of 0.64 % [1]. Congenital CMV infection (cCMV) is the leading non-genetic cause of deafness in children [2] and is also responsible for significant developmental delays in infected children, whether symptomatic or not at birth [3]. Overall ensuing societal costs are estimated to be billions of dollars annually [4, 5], with at least $14.3 million per year for the hospitalization of symptomatic infants in the United States [6].

Primary maternal CMV infection during pregnancy will result in viral transmission to the fetus in 40 % of cases, whereas 1 % of women already infected before pregnancy (known CMV seropositive) will transmit the virus, either through viral reactivation or reinfection with a different strain [7]. However, the severity of fetal complications is highest with maternal primary infection early in pregnancy [8, 9]. Therefore, maternal CMV seronegative status constitutes a major risk factor for congenital CMV disease in children.

Although the role of screening for CMV has been intensely debated in the last decade [10, 11], there remains no consensus on the need to screen all pregnant women, nor on selected populations to target. Recent advances in both diagnostic and therapeutic modalities in managing congenital CMV have also ignited interest in the need for neonatal screening [12–17]. While early treatment may well limit the sequelae of disease in children, prevention remains the cornerstone of efforts to limit the global burden of cCMV disease [18]. Until an effective CMV vaccine becomes available, behavioral interventions may indeed represent the best preventive measure for cCMV. Education on basic hygiene measures to newly pregnant at risk mothers proved to significantly reduce the incidence of maternal infection during pregnancy [19–21]. However, in Canada, there is limited data on which women are actually at risk for primary CMV infection. The first Canadian epidemiologic studies in the 1970s revealed a maternal seronegative status rate of 56–64 % [22–26]. More recent data in selected sub-groups showed a lower rate of 43 % in daycare workers in Montreal [27] and of 45 % in mothers of low birth weight infants in Alberta [28]. Given that socio-demographic factors have greatly changed overtime in Canada, with successive immigration waves, smaller family sizes and new child daycare programs, current data on CMV risk in pregnancy are essential to identify targeted groups for public health interventions.

The primary objective of this study was to determine CMV susceptibility and awareness of CMV disease among pregnant women delivering at Centre Hospitalier Universitaire (CHU) Sainte-Justine, in Montreal, Quebec. Secondary objectives were to determine risk factors for CMV susceptibility and lack of CMV awareness.

## Methods

### Study population

All pregnant women delivering at CHU Sainte-Justine between April and December 2012 were eligible for the study. There are approximately 3500 live births annually at this tertiary care mother-child hospital, which represent 15 % of births in the city of Montreal, which itself represents more than a quarter of births of the Province of Quebec [29]. The cost of medical care for all women is provided by the provincial health plan, under a system of universal health care access.

Inclusion criteria for the study were: 1) Availability of stored first trimester blood in the Sainte-Justine laboratory 2) Age older than 18 years and 3) Capacity to provide written consent in French or English. Mothers whose babies were hospitalized in the neonatal intensive care unit were excluded from the study to prevent additional hospitalization stress. Recruitment occurred on the postpartum ward on 80 pre-specified weekdays, based on staffing availability. Members of the research team (SW and LC) and a research assistant approached mothers before their hospital discharge. During the same interview, they obtained patient consent to participate in the study, administered and simultaneously completed the standard questionnaire described below.

The ethics committee of the Sainte-Justine research center approved the study.

### Serology

Maternal CMV IgG antibody levels were measured on stored first trimester routine blood test using the automated enzyme immunoassay analyzer Triturus (*Diagnostics Grifols, S.A., Barcelona, Spain*). CMV serostatus was defined as negative if a serum titer was < 0.9 U/mL, equivocal between 0.9 and 1.1 U/mL, and positive if >1.1 U/mL. Laboratory results were mailed to the mothers along with an explanatory letter. A pamphlet about preventive measures on CMV transmission was added for seronegative mothers in order to educate them about CMV prevention for subsequent pregnancies.

### Sociodemographic data collection

During the postpartum interview, women were administered a 10-item standardized questionnaire including: knowledge about CMV, knowledge of their CMV status, age, birthplace, age at immigration if foreign born, education level, family revenue, number of children, age of children, number of children who attended daycare, and employment type (See Additional file 1 for full questionnaire). CMV awareness was categorized as CMV aware if women reported awareness by answering yes to either of the following questions: 1) Do you know anything about cytomegalovirus? and 2) Do you know if you are protected against cytomegalovirus? Age was classified into three groups (18–30, 31–35, ≥36 years old). Place of birth of mothers were classified as: Canada, United States of America, Caribbean, South America, Europe, Africa, and Asia. Age at immigration was divided into two groups: younger than 12 years old or older [30]. The three categories for family income (≤$30,000, $31–99,000, and ≥ $100, 000) were based on Statistics Canada classifications [31]. Level of education was categorized into two groups of pre-university and university levels. Occupational exposure was specified as working in child daycare centers, or working in health care facilities, such as hospitals, medical clinics, and long term care institutions, regardless of credentials.

### Statistical analysis

The association between maternal socio-demographic characteristics and CMV seronegative status and CMV awareness were assessed using odds ratios. Multivariable logistic regression was used to adjust for potential confounders identified on univariate analysis and from the literature, including maternal age, birth place, education level, income, number of children, and type of employment. The multivariable analysis was restricted to only those women with complete data on all variables assessed (*n* = 479). All statistical tests were two-sided, and significance was set at a *p* < 0.05. The analysis was conducted using SAS statistical software, version 9.3 (SAS institute, Cary, NC).

## Results

Between April and December 2012 (240 days), 2659 women delivered in the maternity ward of CHU Sainte Justine. Recruitment took place for 80 days during that period. Detailed census of delivered women was collected for a third (27 days between October and December 2012) of the study period, during which time 439 women delivered. Of these women, 314 women were eligible for the study (85 women were excluded as they did not have first trimester serum available, 35 had babies hospitalized in the neonatal intensive care unit, two were minors and three had linguistic barriers). One hundred and fifty-seven (50 %) of these eligible women were not approached for recruitment due to logistic reasons (women not available due to medical interventions, away from the room, busy with other healthcare worker). Of the 157 women approached, nine (5.7 %) refused to participate in the study, resulting in 148 recruited women during the 27-day period. In summary, during the entire 240-day study period, 505 women were successfully recruited over 80 recruitment days. Fourteen of the recruited women were excluded from the final analysis (2 blood samples had insufficient quantity for testing, three laboratory requests were non-conforming, and nine laboratory results were equivocal), leaving 491 study subjects. All of the recruited women completed the questionnaire.

**Fig. 1:**
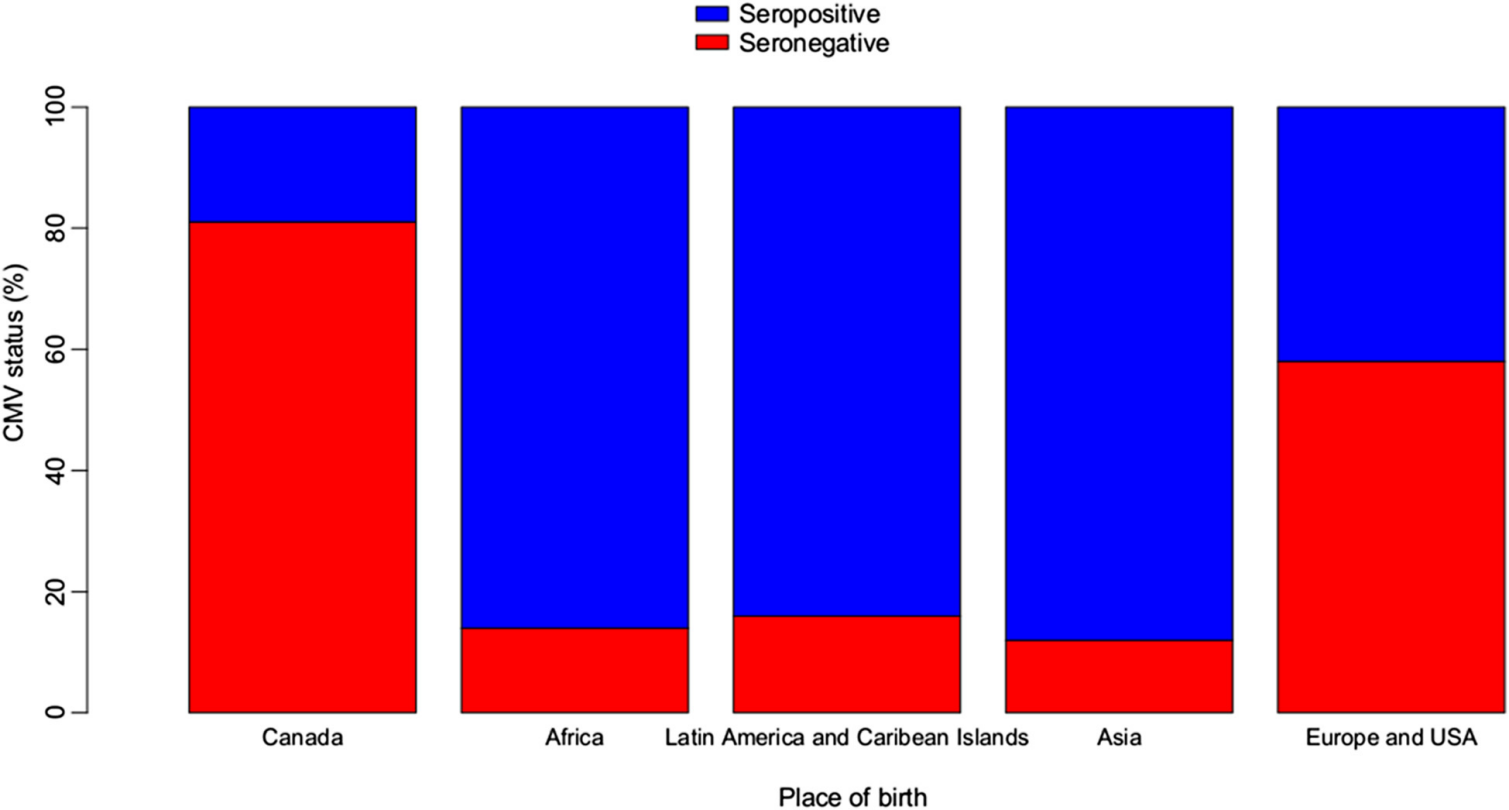
CMV serostatus according to place of birth

Overall, 46 % (*n* = 225) of women were CMV seronegative. The risk of being CMV seronegative according to maternal socio-demographic characteristics is summarized in Table 1. In the unadjusted analysis, significant risk factors for CMV seronegative status included Canadian vs. foreign born women (OR 8.28, 95 % CI 5.45–12.58), high or middle vs. low income (OR 8.13, 95 % CI 4.46–14.85, OR 3.68, 95 % CI 2.10–6.47, respectively), having no children or one other child vs. two children or more (OR 2.08, 95 % CI 1.25–3.47 and OR 2.02, 95 % CI 1.18–3.44, respectively). Compared to those women who did not work outside the home, health care workers had a significantly increased risk of CMV seronegative status (OR 3.35, 95 % CI 1.70–6.62) as did all other trades (OR 2.10, 95 % CI 1.33–3.32), with the exception of daycare workers (OR 1.73, 95 % CI 0.71–4.19). There was no significant effect of age on CMV serostatus. In the multivariate analysis, the only significant risk factors for seronegative status remained Canadian vs. foreign-born women (aOR 6.88, 95 % CI 4.33–10.94), and high or middle vs. low family income (aOR 4.68, 95 % CI 2.09–10.48 and aOR 3.05, 95 % CI 1.54–6.04, respectively). Among foreign-born women, 41 % were from Africa, 27 % from South America or the Caribbean, 24 % from Europe or the United States, and 8 % from Asia. The highest proportion of CMV seronegative foreign-born women was from Europe and the United States (58 %), while the lowest proportion was from Asia (12 %) (Fig. 1). Young age at immigration (≤12 years old) was significantly associated with seronegative status (aOR 4.23, 95%CI 1.28–13.96).

**Table 1:** Maternal CMV serostatus according to socio-demographic characteristics:

	Population	CMV negative	CMV positive	Crude OR (95 % CI)	*p*-value	Adjusted OR† (95 % CI)	*p*-value
	*N* (column %)	*N* (row %)	*N* (row %)			*N* = 479	
	491 (100)	225 (46)	266 (54)				
Age group							
18–30	185 (38)	83 (45)	102 (55)	1.00		0.95	
				(0.63–1.58)	0.98	(0.51–1.76)	0.87
31–35	186 (38)	88 (47)	98 (53)	1.10 (0.69–1.74)	0.69	0.69 (0.39–1.23)	0.21
≥ 36	120 (24)	54 (45)	66 (55)	1		1	
Born in Canada							
Yes	272 (55)	182 (67)	90 (33)	8.28		6.88	
				(5.45–12.58)	<0.0001	(4.33–10.94)	<0.0001
No	219 (45)	43 (20)	176 (80)	1		1	
Education level							
Up to university	188 (38)	76 (40)	112 (60)	1		1	
University	303 (62)	149 (49)	154 (51)	1.43	0.06	1.01	0.96
				(0.99–2.06)		(0.60–1.70)	
Family income^a^ (*N* = 479)							
Low	101 (21)	19 (19)	82 (81)	1		1	
Middle class	228 (48)	105 (46)	123 (54)	3.68	<0.0001	3.05	0.002
				(2.10–6.47)		(1.54–6.04)	
High	150 (31)	98 (65)	52 (35)	8.13	<0.0001	4.68	0.0002
				(4.46–14.85)		(2.09–10.48)	
Other children							
0	229 (47)	113 (49)	116 (51)	2.08	0.01	1.59	0.30
				(1.25–3.47)		(0.66–3.86)	
1	171 (35)	83 (49)	88 (51)	2.02	0.005	1.54	0.19
				(1.18–3.44)		(0.81–2.93)	
≥ 2	91 (18)	29 (32)	62 (68)	1		1	
Daycare							
Yes	218 (44)	93 (43)	125 (57)	1		1	
No	273 (56)	132 (48)	141 (52)	1.26	0.21	1.14	0.74
				(0.88–1.80)		(0.51–2.54)	
Employment							
None	112 (23)	35 (31)	77 (69)	1		1	
DCW	25 (5)	11 (44)	14 (56)	1.73		1.81	
				(0.71–4.19)	0.23	(0.60–5.50)	0.29
HCW	53 (11)	32 (60)	21 (40)	3.35		1.19	
				(1.70–6.62)	0.0005	(0.47–3.03)	0.71
Others	301 (61)	147 (49)	154 (51)	2.10		0.92	
				(1.33–3.32)	0.002	(0.51–1.66)	0.78

^a^low = ≤ 30,000 $/y, middle = 31–99,000 $/y, high = ≥ 100, 000$/y

† OR adjusted for all variables in the table

*DCW* daycare worker, *HCW* health care worker

The association between maternal CMV awareness, and CMV serostatus and socio-demographic factors is summarized in Table 2. Overall, 85 % of women (*n* = 415) were not aware of CMV infection. Among them, 42.4 % (*n* = 176) were CMV susceptible. Significant risk factors for being CMV unaware included foreign vs. Canadian born women (OR 2.06, 95 % CI 1.22–3.49), non-university vs. university educated (OR 2.43, 95 % CI 1.37–4.32), low or middle vs. high income family (OR 5.18, 95 % CI 2.10–12.81 and OR 2.01, 95 % CI 1.18–3.40, respectively), and unemployment or employment in the daycare setting or others trades vs. in the health care setting (OR 64.9, 95 % CI 18.1–232.9, OR 20.6 95 % CI 4.3–96.9 and OR 12.7, 95 % CI 6.6–24.6, respectively). There was no significant effect of age or having children on CMV awareness. In the multivariate analysis however, only employment status remained a significant predictor of CMV unawareness (aOR 85.6, 95 % CI 17.3–421.3, aOR 18.6, 95 % CI 3.6–95.9 and aOR 16.1, 95 % CI 7.9–33.9, for unemployed, daycare workers, and other trades, respectively).

**Table 2:** Maternal CMV awareness according to socio-demographic characteristics

	Population	CMV awareness		Crude OR (95 % CI)	*p*-value	Adjusted OR† (95 % CI)	*p*-value
		Yes	No				
	*N* (column %)	*N* (row %)	*N* (row %)			*N* = 479	
	491 (100)	76 (15)	415 (85)				
CMV status							
CMV negative	225 (46)	49 (22)	176 (78)				
CMV positive	266 (54)	27 (10)	239 (90)				
Age group							
18–30	185 (38)	18 (10)	167 (90)	1.64 (0.81–3.29)	0.17	1.79 (0.74–4.32)	0.20
31–35	186 (38)	40 (22)	146 (78)	0.64 (0.35–1.19)	0.16	0.81 (0.39–1.69)	0.58
≥ 36	120 (24)	18 (15)	102 (85)	1		1	
Born in Canada							
Yes	272 (55)	53 (19)	219 (81)	1		1	
No	219 (45)	23 (11)	196 (89)	2.06 (1.22–3.49)	<0.01	1.81 (0.92–3.55)	0.08
Education level							
Up to university	188 (38)	17 (9)	171 (91)	2.43 (1.37–4.32)	<0.003	1.40 (0.66–2.97)	0.38
University	303 (62)	59 (19)	244 (81)	1		1	
Family income^a^ (*N* = 479)							
Low	101 (21)	6 (6)	95 (94)	5.18 (2.10–12.81)	<0.001	0.89 (0.28–2.86)	0.84
Middle class	228 (48)	32 (14)	196 (86)	2.01 (1.18–3.40)	<0.01	1.02 (0.52–2.04)	0.95
High	150 (31)	37 (25)	113 (75)	1		1	
Other children							
0	229 (47)	35 (15)	194 (85)	1		1	
1	171 (35)	32 (19)	139 (81)	0.78 (0.46–1.33)	0.36	0.88 (0.27–2.88)	0.93
≥ 2	91 (18)	9 (10)	82 (90)	1.64 (0.76–3.57)	0.21	2.67 (0.63–11.41)	0.18
Daycare							
Yes	218 (44)	35 (16)	183 (84)	1		1	
No	273 (56)	41 (15)	232 (85)	1.08 (0.66–1.77)	0.75	0.84 (0.26–2.75)	0.77
Employment							
None	112 (23)	3 (3)	109 (97)	64.9 (18.1–232.9)	<0.0001	85.6 (17.3–421.3)	<0.0001
Others	301 (61)	37 (12)	264 (88)	12.7 (6.6–24.6)	<0.0001	16.1 (7.6–33.9)	<0.0001
DCW	25 (5)	2 (8)	23 (92)	20.6 (4.3–96.9)	0.0001	18.6 (3.6–95.9)	0.0005
HCW	53 (11)	34 (64)	19 (36)	1		1	

^a^low = ≤ 30,000 $/y, middle = 31–99,000 $/y, high = ≥ 100, 000$/y

† OR adjusted for all variables in the table except CMV status

*DCW* daycare worker, *HCW* health care worker

## Discussion

In this single center observational study from the cosmopolitan city of Montreal, Quebec, we identified a number of risk factors for CMV seronegative status and lack of CMV awareness among post-partum women. While overall CMV susceptibility among pregnant women from the same hospital has decreased from a reported 63.3 % [25] in 1972 to 46 % in the present study, Canadian born women continue to be at increased risk of CMV seronegative status when compared to foreign born. In that previous study, 73 % of Canadian mothers were seronegative vs. 29 % of Italian mothers, which is comparable to the current 66 % susceptibility rate in Canadian born vs. 20 % in foreign mothers in the present study. The overall decrease in the number of CMV seronegative pregnant women is likely related to the smaller proportion of Canadian born women, due to migratory trends in the province of Quebec since the end of the 1970s [29]. Family income was identified as another factor for CMV seronegative status, with women of higher family income at increased risk of CMV seronegative status. These findings were also compatible with the recent Australian study that highlighted a marked socioeconomic gradient in CMV seroprevalence and its positive association with congenital CMV [32]. Our overall proportion of CMV seronegative pregnant women is similar to that reported in other wealthy countries such as Australia (43 %), France (48 %), and United States (28–49 %) [32].

In contrast with previous studies [33, 34], risk factors that were not significant for seronegative status in the present study included age, level of education, number of children, and attendance at daycare. While this may in part be due to our relatively small sample size, we suspect that socio-demographic trends unique to Canada, and specifically to the province of Quebec, may provide further explanation.

Education level was not predictive of CMV susceptibility potentially because our population was highly educated (61 % had a university degree) independently of the country of birth. In fact, Canada has the highest proportion (64.1 %) of adult population with post-secondary education among developed countries and the proportion of recent immigrants with a university degree is twice as high as among Canadian born fellows [35].

Family size and subsidized day care centers programs are also recent changes that might have influenced the epidemiology of CMV in the province of Quebec. Family size in Canada decreased from 3.7 in 1971 to 2.5, and even to 2.3 persons in Quebec, in 2011 [36]. With an increase in the number of subsidized child daycare programs across Canada, childcare spaces doubled from 1992 to 2004, of which 43 % were in Quebec (although this province represents only 22 % of the Canadian population) [36]. Among the 262 studied women who had children previously, 83 % had children with day care exposure. These two risk factors were therefore too correlated to measure the independent effects from children exposure at home and their daycare attendance on maternal seronegative status.

Finally, we did not see an increased risk of seronegative status among daycare workers. While they were only 25 mothers in that category, their seronegativity rate was comparable to that of the whole cohort. This is similar to the result of a previous study of daycare workers in Montreal, where 43 % were seronegative, although 69 % were Canadian born [27]. Daycare workers in Toronto had a lower seronegativity rate (33 %) but those seronegative workers experienced a high rate of seroconversion (12.5 % in 1 year) [37].

Perhaps most concerning is that while 46 % of mothers studied were seronegative and thus susceptible to primary infection during pregnancy, only 15 % of mothers (and 22 % of the seronegative ones) were aware of CMV. Even among healthcare workers, only 64 % were aware of CMV in our study. This level of awareness about CMV was similar to the 14–25 % rate reported in United States [38, 39], and the 12.5 % among Dutch pregnant women [40]. Interestingly, although CMV is the most common congenital infection, Dutch mothers seemed much more aware of other congenital infections like toxoplasmosis and listeriosis. Identifying women with a lack of CMV awareness is important so as to target appropriate public health interventions. Lack of knowledge may lead to more risky behaviors and increase risks of infection. It has been well demonstrated that seronegative mothers with a child less than 36 months of age attending daycare who already knew that they were pregnant were more motivated to alter behavior than mothers attempting pregnancy. In that study, counseling pregnant women was 85 % effective in reducing risk of CMV acquisition [41]. Indeed, according to the 2002 statement of the American College of Obstetrics and Gynecology, the greatest impact to reduce CMV diseases is educating pregnant women about preventive measures [42]. Yet, less than half of US obstetricians had mentioned them to their patients in the 2008 National Survey [43]. Other than health care workers, most of our participants were unaware about CMV, even those working in high-risk setting such as the daycare. Given the few CMV aware women, our sample size may have been too small to discern that influence.

The limitations of the present study are that it was a single center study, which may not be representative of the larger Canadian population. While the proportion of foreign-born mothers in our population was representative of the metropolitan Montreal population [29] it does not reflect the rest of the province of Quebec, which has lower immigrant populations [44], or Western Canada, which counts immigrants mostly from Asia and India. Moreover, CHU Sainte-Justine is a francophone referral hospital, and thus mothers from the First Nations and the Asian continent (more likely to be English speaking) are likely underrepresented. Furthermore, the level of wealth of participants in our study is higher than that described in the general population of Canada, which may make it difficult to generalize our results. Finally, our sample size may have been too small to detect differences in the socio-demographic risk factors for CMV seronegative status and awareness, given the large number of explanatory variables per category. Larger, more representative population level studies are needed to identify more precisely these risk factors and target groups for anticipatory behavioral intervention.

## Conclusions

In conclusion, in the present study, we identified a high proportion of CMV seronegative status and unawareness among pregnant women. Given that there is no current consensus on the role of CMV screening of pregnant women, primary prevention through education of at risk women about CMV transmission and basic hygiene preventive measures could potentially have a major public health impact. Moreover, increased education of health care workers, who themselves may lack CMV awareness, could help reinforce these education measures. In this respect, collaboration with other large mother-child centers in the province of Quebec and other provinces across Canada are needed to improve the reproducibility and generalizability of our findings and to develop public health interventions.

## Additional file

Additional file 1: CMV questionnaire. (DOC 45 kb)
