# Supplementary material for: Awareness of cytomegalovirus and risk factors for susceptibility among pregnant women, in Montreal, Canada
Source: BMC Pregnancy Childbirth. 2016 Mar 15;16:54. doi: 10.1186/s12884-016-0844-9 (PMC4793542; doi:10.1186/s12884-016-0844-9)
Supplement: Additional file 1: — CMV questionnaire. (DOC 45 kb) [file 12884_2016_844_MOESM1_ESM.doc]

QUESTIONNAIRE

1. Identification number : ___________________
2. Do you know anything about cytomegalovirus? □ Yes □ No
3. Do you know if you are protected against cytomegalovirus? □ Yes □ No
4. Date of Birth (year) : ______________
5. Were you born in Canada? □ Yes □ No

- If Yes : Which origin are you ?

□ Canadian

□ First Nations

□ Other

- If No : Arrival date in Canada (year) __________________

Origin : □ North Africa

□ Sub-Saharan Africa

□ Caribbean Islands

□ Latin America

□ United States of America

□ Europe

□ Asia

1. Education level : □ Not completed high school

□ High school

□ College

□ University

1. Family revenue: □ <10 000 $

□ 10-29 999 $

□ 30-59 999 $

□ 60-99 999 $

□ ≥100 000 $

1. How many children live with you at home (excluding the newborn)?

□ 0

□ 1

□ 2

□ 3

□ 4

□ more than 4

1. How old are your children?

□ 1-2 years old

Are they going to daycare? □ Yes □ No

□ 2-5 years old

Are they going to daycare? □ Yes □ No

□ More than 5 years old

Did they ever go to daycare? □ Yes □ No

If yes: At what age? _______________________

1. Are you working ?

□ No

□ Child daycare center

□ Healthcare center

□ other
